# Enrichable consortia of microbial symbionts degrade macroalgal polysaccharides in *Kyphosus* fish

**DOI:** 10.1128/mbio.00496-24

**Published:** 2024-03-27

**Authors:** Aaron Oliver, Sheila Podell, Linda Wegley Kelly, Wesley J. Sparagon, Alvaro M. Plominsky, Robert S. Nelson, Lieve M. L. Laurens, Simona Augyte, Neil A. Sims, Craig E. Nelson, Eric E. Allen

**Affiliations:** 1 Center for Marine Biotechnology and Biomedicine, Scripps Institution of Oceanography, University of California San Diego, La Jolla, California, USA; 2 Marine Biology Research Division, Scripps Institution of Oceanography, University of California San Diego, La Jolla, California, USA; 3 Daniel K. Inouye Center for Microbial Oceanography: Research and Education, School of Ocean and Earth Science and Technology, University of Hawai’i at Mānoa, Honolulu, Hawaii, USA; 4 National Renewable Energy Laboratory, Golden, Colorado, USA; 5 Ocean-Era Inc., Kailua-Kona, Hawaii, USA; 6 Center for Microbiome Innovation, University of California San Diego, La Jolla, California, USA; 7 Department of Molecular Biology, School of Biological Sciences, University of California San Diego, La Jolla, California, USA; University of California, Irvine, Irvine, California, USA

**Keywords:** *Kyphosus*, fish gut microbiome, macroalgal polysaccharides, sulfatase

## Abstract

Coastal herbivorous fishes consume macroalgae, which is then degraded by microbes along their digestive tract. However, there is scarce genomic information about the microbiota that perform this degradation. This study explores the potential of *Kyphosus* gastrointestinal microbial symbionts to collaboratively degrade and ferment polysaccharides from red, green, and brown macroalgae through *in silico* study of carbohydrate-active enzyme and sulfatase sequences. Recovery of metagenome-assembled genomes (MAGs) from previously described *Kyphosus* gut metagenomes and newly sequenced bioreactor enrichments reveals differences in enzymatic capabilities between the major microbial taxa in *Kyphosus* guts. The most versatile of the recovered MAGs were from the *Bacteroidota* phylum, whose MAGs house enzyme collections able to decompose a variety of algal polysaccharides. Unique enzymes and predicted degradative capacities of genomes from the *Bacillota* (genus *Vallitalea*) and *Verrucomicrobiota* (order *Kiritimatiellales*) highlight the importance of metabolic contributions from multiple phyla to broaden polysaccharide degradation capabilities. Few genomes contain the required enzymes to fully degrade any complex sulfated algal polysaccharide alone. The distribution of suitable enzymes between MAGs originating from different taxa, along with the widespread detection of signal peptides in candidate enzymes, is consistent with cooperative extracellular degradation of these carbohydrates. This study leverages genomic evidence to reveal an untapped diversity at the enzyme and strain level among *Kyphosus* symbionts and their contributions to macroalgae decomposition. Bioreactor enrichments provide a genomic foundation for degradative and fermentative processes central to translating the knowledge gained from this system to the aquaculture and bioenergy sectors.

**IMPORTANCE:**

Seaweed has long been considered a promising source of sustainable biomass for bioenergy and aquaculture feed, but scalable industrial methods for decomposing terrestrial compounds can struggle to break down seaweed polysaccharides efficiently due to their unique sulfated structures. Fish of the genus *Kyphosus* feed on seaweed by leveraging gastrointestinal bacteria to degrade algal polysaccharides into simple sugars. This study reconstructs metagenome-assembled genomes for these gastrointestinal bacteria to enhance our understanding of herbivorous fish digestion and fermentation of algal sugars. Investigations at the gene level identify *Kyphosus* guts as an untapped source of seaweed-degrading enzymes ripe for further characterization. These discoveries set the stage for future work incorporating marine enzymes and microbial communities in the industrial degradation of algal polysaccharides.

## INTRODUCTION

The *Kyphosus* genus of herbivorous fish, commonly referred to as nenue or rudderfish, graze primarily on macroalgae (1). *Kyphosus* fish serve important ecological roles by controlling algal cover in Indo-Pacific (2) and Caribbean coral reefs (3), thereby mediating coral-algal competition, overall coral growth, and benthic community composition (4). Their diverse diet includes macroalgae from the three major taxonomic groups: Rhodophyta (red), Chlorophyta (green), and Ochrophyta (brown) (1). Polysaccharides constitute as much as 60% of macroalgal cells by weight (5) and serve roles in both cell structure and energy storage (6). The complex network of linkages in structural polysaccharides resists degradation from chemical and enzymatic stressors and serves as a physical defense mechanism for algal cells (7).

Algal polysaccharides differ from common polysaccharides found in land plants due to the addition of sulfate ester groups (8). Structural polysaccharides from red algae include agar, carrageenan, porphyran, and xylan, which all contain such sulfate groups (9). Brown algae contain the sulfated polysaccharide fucoidan for structure as well as unsulfated alginate as a storage polysaccharide (9). Green algae contain sulfated polysaccharides such as xylan and ulvan but also contain large amounts of unsulfated cellulose common in land plants (9). Algal polysaccharides are depolymerized primarily through the enzymatic activity of bacterial glycoside hydrolases (GHs) and polysaccharide lyases (10), two classes of carbohydrate-active enzymes (CAZymes) (11). Sulfated polysaccharides are particularly recalcitrant to digestion because an additional enzyme class, the sulfatases, is necessary for complete degradation. Full enzyme pathways for the breakdown of various algal polysaccharides have been proposed (9, 12) that include both required CAZyme and sulfatase activities. However, not all algal polysaccharides have well-defined degradation pathways or unique associated CAZymes that enable a high-level connection between gene presence and catabolized substrates. Likewise, sulfatase classes within the SulfAtlas database (13) are primarily classified based on evolutionary history rather than substrate specificity or enzymatic activity, so our ability to evaluate pathway completeness *in silico* is limited.

Once complex carbohydrates are broken into subunits by CAZymes and sulfatases, they are utilized by gut microbiota in fermentation reactions to produce short-chain fatty acids (SCFAs) (14). The SCFAs acetate, propanoate, and butyrate have been previously measured in high quantities in *Kyphosus* hindguts (15) and are utilized by the host fish for energy (16). Previous work has suggested correlations between SCFA profiles and bacterial composition (15), but there is no genomic work in algivorous fish pinpointing which microbiota contribute to host nutrition in this way and what pathways are utilized to produce these essential SCFAs.

Our overall understanding of the role of gut microbiota in digestion is still limited in most fishes (17), including *Kyphosus*, in part due to a focus on community composition and diversity rather than function. The genetic study of *Kyphosus* gut symbionts has been limited to 16S rRNA (15, 18) and metabolomic (18) investigations until the incorporation of shotgun metagenomics in a few recent studies (19, 20). What functional profiling has been done in fish guts often relies on extrapolation from amplicon-based taxonomic distributions (21–24), and no study has yet generated a large collection of metagenome-assembled genomes (MAGs) from an algivorous fish gut. A *de novo* genomic investigation of *Kyphosus* symbionts has the potential to reveal degradative capacities that cannot be extrapolated from taxonomic lineage or relatedness to database representatives.

Discoveries from better-studied human gut and terrestrial herbivore systems provide suggestions for how *Kyphosus* symbionts might gain and use such gene pathways. Human gut bacteria have acquired enzymes that degrade sulfated algal polysaccharides through horizontal gene transfer (25, 26). Horizontal gene transfer of antibiotic resistance genes has also been observed in fish gut biofilms (27), but this phenomenon has not yet been reported for carbohydrate-active enzymes in any fish gut symbiont microbe. Once acquired, CAZymes and sulfatases potentially originating from one or multiple organisms may then decompose algal polysaccharides in complex, stepwise pathways. A cooperative division of labor strategy, in which partial breakdown products from one bacterial population serve as a degradative substrate for other bacteria in the community, has been proposed to occur in human gut microbiota (28) and has been suggested as a way to improve polysaccharide degradation in engineered communities (29). The degree to which collaboration may occur in the herbivorous fish gastrointestinal tract remains unknown.

Exploring functional diversity not only improves our understanding of herbivorous fish digestion but may also enable concrete applications in the fields of aquaculture and bioenergy. Most aquaculture is currently sustained through compound feeds that are composed of fishmeal and fish oils from wild-caught fish (30). Although innovations in aquaculture feed have lowered the trophic levels of captive carnivorous fish and improved overall feed efficiency (31), concerns about sustainability and food security remain. Wan et al. (32) argue that the discovery of efficient methods to degrade complex polysaccharides and enhance nutrient digestibility is a key knowledge gap and barrier limiting macroalgae inclusion into commercial aquafeeds (32). Macroalgal feed additives are also known to counteract methanogenesis in terrestrial ruminants (33) and thus can be applied to reduce methane emissions from livestock husbandry. However, deficiencies in ruminant microbiome digestive capacities may influence the future development and long-term success of seaweed dietary supplementation strategies. Research on *Kyphosus* symbionts and their enzymes can inspire commercializable and scalable methods to break down these barriers in the industry.

Innovations exploiting the experimental propagation of enrichment cultures with *Kyphosus* symbionts can harness these microbial communities for further study and experimentation with commercial outputs in the bioenergy sector as well as the development of macroalgal feed supplements. While a few bacterial isolates have been recovered and sequenced from kyphosid guts (34), no previous study has enriched entire communities from these fishes to investigate their hydrolytic and fermentative capabilities. Hydrolysis of carbohydrates, proteins, and lipids into their monomeric components is a key step in biogas and bioethanol production from macroalgae (35, 36), and the degradation of algal polysaccharides is often the rate-limiting step in anaerobic digestion (37). Milledge et al. (38) call for future studies to look beyond commercially available enzymes to discover candidates that can more efficiently degrade algal polysaccharides (38). The *Kyphosus* gut, with its understudied functional diversity and degradative pathways, offers an untapped source of such enzyme and inoculum candidates.

This study leverages metagenome-assembled genomes from *Kyphosus vaigiensis*, *Kyphosus cinerascens*, and *Kyphosus hawaiiensis* gut symbionts and inoculated bioreactor enrichments to connect whole-genome degradative potential of algal polysaccharides to accurate taxonomic lineages and functional roles. The addition of genomes from bioreactor enrichments explores leveraging the metabolic capacities of *Kyphosus* gut consortia in industrial processes. This work extends previous studies of taxonomic-level biogeography (18) and contig-level gene associations (15, 20) in this system using high-quality MAGs, which enables differentiation between processes that can potentially be executed within a single cellular compartment (individual microbial species/population) and those likely to require cooperative action by multiple cells from different species (community impacts). Discoveries in this study provide the foundation for genome-level understanding of microbial contributions to herbivorous fish digestion and beget future investigations to apply these findings toward applications in the aquaculture and bioenergy sectors.

## MATERIALS AND METHODS

### Sample description and metagenomic assembly

DNA was extracted from liquid samples from 10 anaerobic bioreactors inoculated with gut content from either “Fish 6” (*K. cinerascens*) or “Fish 7” (*K. hawaiiensis*; Table S1) using methods previously described (18) and propagated to enrich degradative properties. Samples were taken 9–10 days after inoculation and incubation at 30°C. A 35 psu Artificial Sea Water (ASW) solution was prepared by dissolving and autoclaving 40 g/L of Instant Ocean sea salts (Instant Ocean, Spectrum Brands, Blacksburg, VA). The Basal Salts Medium solution was then prepared by dissolving and autoclaving 90 mM MgSO_4_, 6 mM K_2_CO_3_, 6 mM CaCO_3_, 20 mM MgCO_3_, and 1/10 of the final volume of ASW in ultrapure water. Anoxic cultures of 50 mL were processed in a portable anaerobic chamber containing sterile Basal Salts Medium in 150 mL serum bottles, crimp sealed with a rubber septum. These ionic concentrations were selected to simulate the estimated osmolarity of seawater as it passes through the midgut and hindgut (39). Approximately 1 g of fish gut section contents were placed in the bottles along with the indicated substrate (Table S1) and sealed, with no additional feedstock added before sequencing. Substrate selection was focused on polysaccharides and algal species of particular relevance to bioenergy and bioproduct production.

Samples were sequenced using Illumina NovaSeq 6000 technology (Illumina, San Diego, CA). Read trimming was performed using Trimmomatic v. 0.36 (40) with the following parameters: adapter-read alignment settings 2:30:10, LEADING:10, TRAILING:20, HEADCROP:12, SLIDINGWINDOW:4:15, and MINLEN:200. Taxonomic composition of metagenomic reads was determined using Kraken v. 2.0.9 (41), with taxonomic assignment using a protein database based on all amino acid sequences in the NCBI nr database (42) as of April 2022. Cleaned reads were assembled in metaSPAdes v. 3.13 (43) with a minimum contig retention size of 2,000 nucleotides.

### Gene calling and functional annotation

Gene boundaries were predicted using prodigal v. 2.6.2 (44) and annotated using prokka v. 1.12 (45). Genes were assigned to CAZy classes from the dbCAN HMMdb v. 10 database (46) based on the CAZy database (11) and to sulfatases classes from the SulfAtlas v. 2.3 database (13), using methods previously described (20). Signal peptides were identified using SignalP v. 6 (47) with default parameters. Additional enzyme classes were annotated with KofamKOALA (48).

Enzyme novelty was evaluated using DIAMOND blastp (49) searches against the NCBI nr database (42) as of April 2022. Some CAZyme classes were grouped into the category of “peptidoglycanases” using the division proposed by López-Mondéjar et al. (50). Distributions of annotated proteins were compared to free-living relatives from the OceanDNA database (51).

### Metagenomic binning and biosynthetic gene cluster prediction

Metagenomic binning was performed from both newly assembled bioreactor metagenomes described above and *in vivo* gut metagenomes from *K. vaigiensis*, *K. cinerascens*, and *K. hawaiiensis* lumen contents previously described in Podell et al. (20). Lumen contents were used to maximize microbial biomass while reducing the amount of recovered eukaryotic host DNA. Binning was done through MetaWRAP v. 1.3.2 (52) with a minimum completeness cutoff of 0.7 and a maximum contamination cutoff of 0.05 as determined by CheckM v. 1.0.12 (53). MAG taxonomy was determined using GTDB-Tk v. 1.5.1 (54) with release 202 of the Genome Taxonomy Database (55).

Viral contigs and prophages were identified using DeepVirFinder v. 1.0 (56) using a *q*-score cutoff of 0.94. Viral sequence completeness was determined using CheckV v. 1.5 (57), retaining only regions marked as “high-quality” or “complete.” Viral sequences were assigned to host taxonomies using the software VPF-Class (58).

Biosynthetic gene clusters (BGCs) were predicted for each MAG using antiSMASH v. 6.1 (59). Predicted products and BGC classes were annotated using BiG-SLiCE v. 1.1.1 (60). Gene cluster distances were calculated using the BiG-FAM webservice v. 1.0.0 (61), using a novelty distance cutoff of 900 following previous studies (61–63). Short-chain fatty acid gene clusters were annotated using gutSMASH v. 5.0.0 (64).

### Phylogenomics and enzyme phylogenetics

A phylogenetic tree of MAGs was generated using PhyloPhlAn v. 3.0.2 (65) using a concatenated universal set of 400 marker genes (66). MAGs containing at least 100 marker genes underwent concatenated alignment using MAFFT v. 7.505 (67). The phylogenetic tree was built using RaxML v. 8.2.12 (68) and visualized using R v. 4.2.0 (69) packages treeio v. 1.20.0 (70), ggtree v. 3.4.0 (71), and ggtreeExtra v. 1.6.0 (72).

Multiple sequence alignments for genes belonging to CAZy class GH86 were made using MUSCLE v. 3.8.31 (73) and visualized using the R package ggmsa v. 1.2.0 (74). Gene trees were created using FastTree v. 2.1.10 (75). Additional reference genes were included in the tree based on DIAMOND blastp matches to the NCBI nr database as of April 2022. Protein domains were analyzed with the CDD webservice (76). Three-dimensional protein structures for CAZymes were predicted using ColabFold v. 1.3.0 (77) and visualized using ChimeraX v. 1.3 (78). Residue conservation was visualized using the WebLogo (79) webservice.

## RESULTS

### A (meta)genome catalog of enrichable symbionts in the *Kyphosus* gut

**Fig 1:**
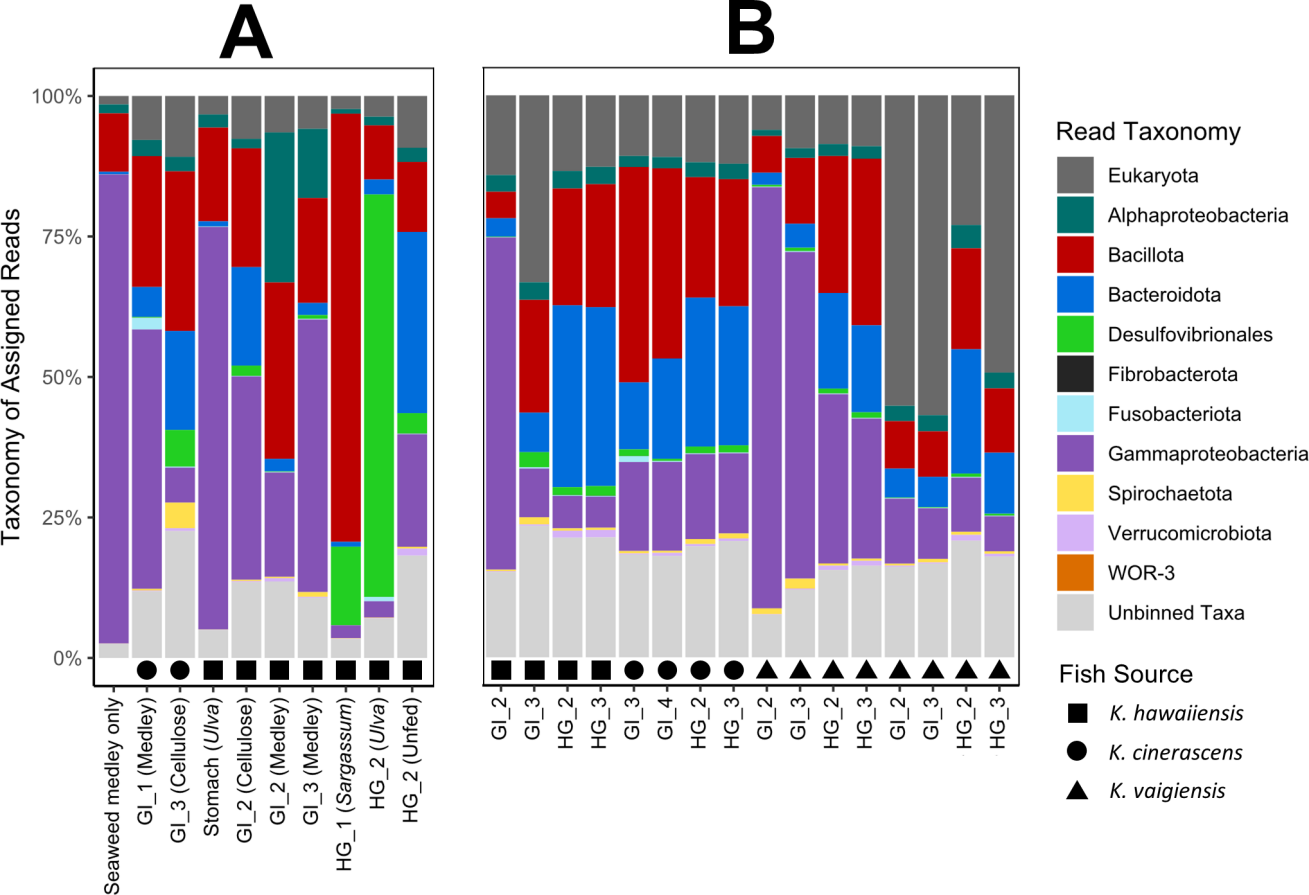
Taxonomic distribution of enrichment and fish gut samples. Unassembled metagenomic reads were classified using Kraken2. (**A**) Enrichment samples are labeled with inoculant fish taxa, gut region, and bioreactor feed. (**B**) Wild fish gut metagenomic samples previously assembled by Podell et al. (20). Shapes along the x-axis denote the species of *Kyphosus* whose gut was either (**A**) used as the inoculant or (**B**) directly sequenced. Abbreviations: GI, midgut; HG, hindgut; medley, a combination of *Ulva*, *Sargassum*, and *Agardhiella* seaweed.

New data derived from *K. cinerascens* and *K. hawaiiensis* enrichment cultures expand the diversity of previous *K. cinerascens*, *K. hawaiiensis*, and *K. vaigiensis* gut metagenomes (20). This more complete catalog of *Kyphosus* gut microbiota provides additional details on the metabolic potential of taxa that were rare in the *in vivo* gut metagenome samples and highlights potential challenges in harnessing gastrointestinal microbiota for industrial processes. The fish inoculum species, gut location, and feedstock that were combined to establish each enrichment sample are described in Table S1. The taxonomic classification of unassembled metagenomic reads revealed high-level consistency at the phylum level between the *in vivo* gut microbiomes (20) and enrichment samples (Fig. 1). *Bacillota*, *Bacteroidota*, and *Gammaproteobacteria* constitute the dominant bacterial lineages in most samples, although the *Desulfovibrionales* order (phylum *Thermodesulfobacteriota*) was highly abundant in two enrichment samples.

Seventy-four medium- and high-quality MAG bins were obtained from newly assembled enrichment metagenomes, along with 137 new bins from previously described wild fish gut metagenomes (Fig. S1). These MAGs all met the minimum of 70% completion and a maximum of 5% redundancy standards (80). Assembly statistics for enrichment metagenomes are shown in Table S2, and MAG summary metrics outlined by the Genomic Standards Consortium (80) are provided in Table S3. Consistent with the unassembled read-based taxonomic profiles of the metagenomes, most MAGs were assigned to the phyla *Bacillota* (78 MAGs), *Bacteroidota* (72 MAGs), the class *Gammaproteobacteria* (31 MAGs), or the order *Desulfovibrionales* (13 MAGs), along with phylum *Verrucomicrobiota* (6 MAGs). The enrichments provide information on microbial members that were not as abundant in the fish gut metagenomes and vice versa. In one example, bins containing the *Verrucomicrobiota* order *Kiritimatiellales* were recovered in *K. cinerascens* gut samples but not in enrichment metagenomes. These dissimilarities were also reflected in nucleotide similarities, as only 9 of the 74 (12%) enrichment MAGs match MAGs generated from *in vivo* fish gut metagenomes at the species level. Enrichment samples averaged approximately 6% eukaryotic reads, while adult fish gut samples averaged 13%, and juvenile fish gut samples averaged 46%, possibly due to the technical limitations of collecting ample microbial biomass from smaller fish.

Viral and archaeal sequences comprised less than 0.5% of all unassembled metagenomic reads, with 69 viral contigs and 3 prophages identified as either high quality or complete. Within these viral elements, 30 auxiliary metabolic genes found on potential prophage regions were annotated as CAZymes and 13 as sulfatases, suggesting a potential role for viral dissemination of these genes across the bacterial community. The taxa *Bacillota*, *Bacteroidota*, and *Gammaproteobacteria* were the most frequently predicted viral hosts (Table S4), which is consistent with the taxonomic abundances of classified unassembled metagenomic reads and recovered MAGs. Despite the presence of numerous auxiliary metabolic genes annotated as mediating more general polysaccharide degradation, none of the viral sequences we detected appeared to specifically target large, complex sulfated macroalgal polysaccharides.

### Genome capacities reveal metabolic specialization among gut symbionts of *Kyphosus* fish

**Fig 2:**
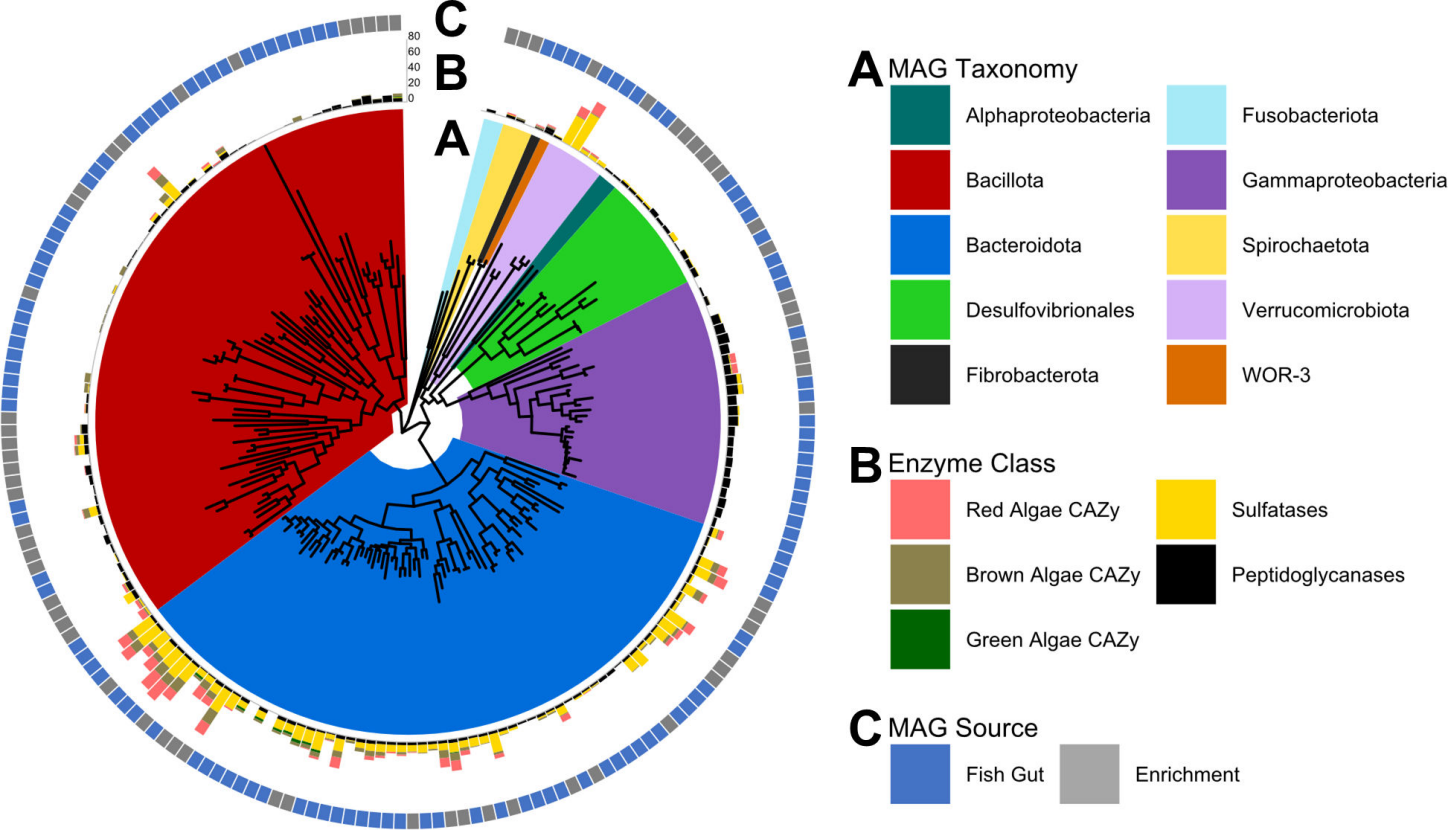
Genomic CAZyme distributions reveal connections between metabolic strategies and taxonomic lineage. (**A**) The gene tree shows a concatenated alignment of 400 PhyloPhlAn universal marker genes for each recovered MAG, with branches colored by assigned MAG taxonomy. (**B**) The inner ring displays genomic gene counts for sulfatases and carbohydrate-active enzymes that specifically target algal polysaccharides or peptidoglycans. (**C**) Environmental source of each MAG.

The distribution of CAZymes and sulfatases was correlated with the phylogeny of fish gut and enrichment MAGs, as shown using a concatenated marker gene tree (Fig. 2A). This assessment revealed that among the MAGs generated in this study, the *Bacteroidota* genomes contained the majority of CAZymes and sulfatases (Fig. 2B). Algal degradation-specific CAZyme-rich genomes among the MAGs from other phyla were restricted either to a single order, *Kiritimatiellales* (*Verrucomicrobiota*), or a single genus, *Vallitalea* (*Bacillota*). Recovered *Gammaproteobacteria* and *Desulfovibrionales* genomes lacked enzymes required for digesting sulfated algal polysaccharides despite the relatively high abundance of these taxonomic groups in classified unassembled reads and the recovered MAGs. However, the *Gammaproteobacteria* MAGs contained more peptidoglycanases than other taxa, suggesting a potential niche in digesting alternative dietary components. This analysis also showed that CAZymes targeting ulvan, a green algal polysaccharide, were less prevalent among the symbiotic MAGs associated with wild fish than CAZymes targeting red and brown algae-associated polysaccharides (Fig. 2B), consistent with previous results quantifying relative amounts of these algae types consumed by the *Kyphosus* fish included in this study (20). The most abundant phyla yielded binned MAGs from both *in vivo* and enrichment samples (Fig. 2C).

A search was performed for genes involved in mannitol metabolism to determine whether this sugar alcohol, known to be abundant in brown algae, might be used for fermentation. In support of this hypothesis, genes predicted to encode mannitol 2-dehydrogenases, mannitol-1-phosphate 5-dehydrogenases, mannitol-specific phosphotransferase system (PTS) enzymes, and mannitol operon repressors were detected in both MAGs and metagenomes from natural fish gut samples as well as enrichment cultures (Table S5). This metabolic potential was not lineage-specific, as MAG representatives from *Bacteroidota*, *Bacillota*, *Gammaproteobacteria*, and *Verrucomicrobiota* all contained these genes, and 22% of our recovered MAGs contained at least one of the two major enzyme classes thought to contribute to mannitol to fructose conversion in *Kyphosus* guts (19). Even though not all genes were present in all samples, it was not possible to conclude whether differences between samples might be significant due to unavoidable variability in overall community complexity, assembly efficiency, MAG completeness, and uneven representation of less abundant taxa.

**Fig 3:**
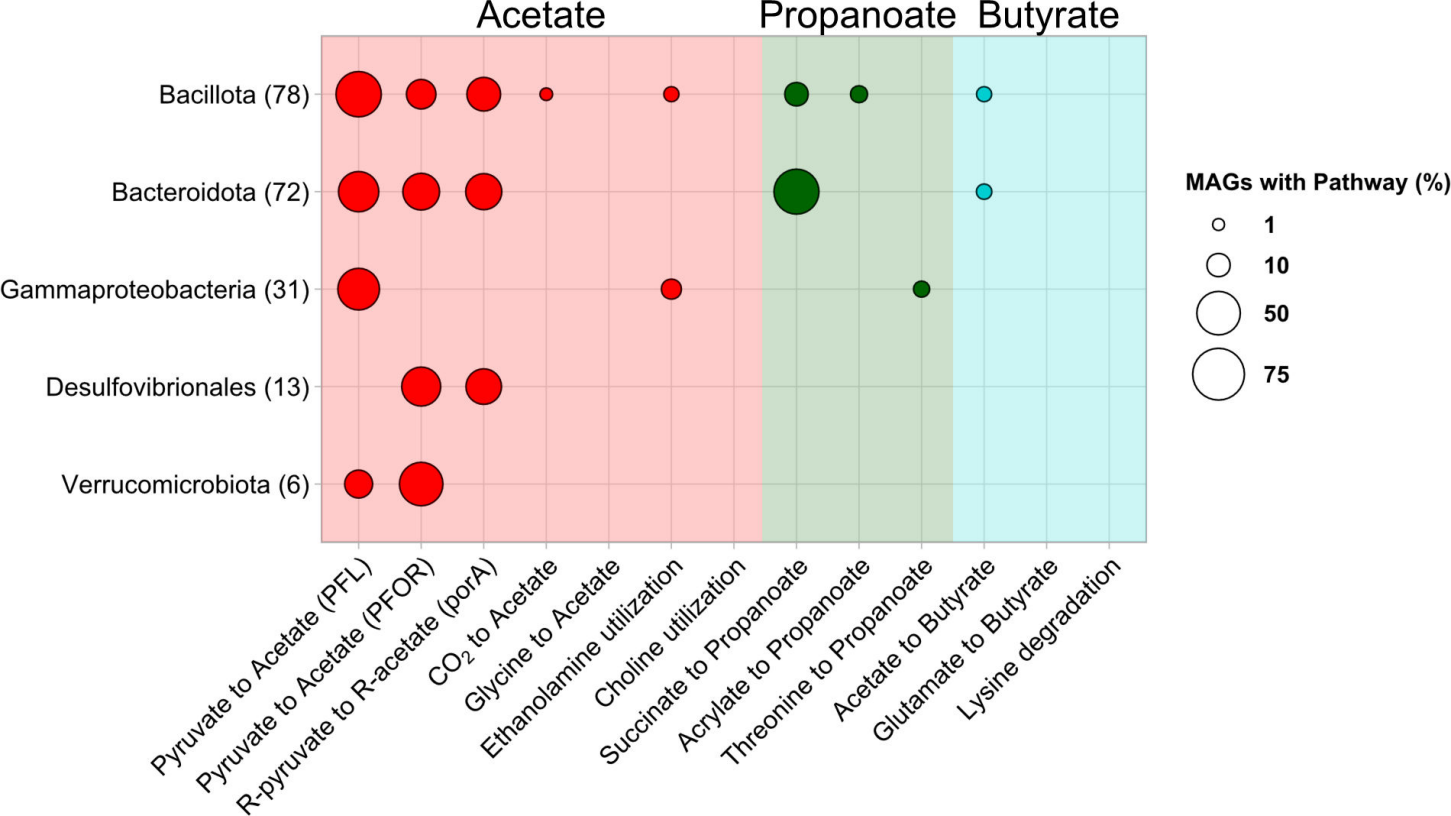
*Kyphosus* gut symbionts use diverse metabolic strategies to produce SCFAs. The bubble plot displays the presence of pathways in MAGs from different taxonomic groups as determined by gutSMASH. Circles increase in size based on the proportion of binned genomes that are annotated with each pathway. Bubbles and chart backgrounds are colored based on their association with the production of the fatty acids acetate (red), propanoate (green), or butyrate (cyan). Counts next to taxonomic names denote the number of MAGs analyzed from that taxa.

An assessment of SCFA production gene pathways of recovered MAGs using gutSMASH (64) revealed that most of the *Kyphosus* gut symbiotic taxa (67% of fish gut MAGs and 77% of enrichment MAGs) can potentially contribute to host nutrition through the production of SCFAs (Fig. 3). One hundred thirty-nine genomes from analyzed kyphosid fish gut microbial communities contained pathways for producing acetate, but only six genomes contained pathways for butyrate production. The pyruvate formate lyase and pyruvate:ferredoxin oxidoreductase pathways were the most abundant overall, present in 126 MAGs, while *Bacteroidota* contained the most gene clusters (39) related to propanoate production.

The overall prevalence of acetate production pathways was lower than that previously reported in human gut microbiota (81). The total absence of some alternate fermentation pathways from our MAGs, such as choline utilization, suggests that those processes are not core to dominant members of the *Kyphosus* gut microbiome. Only one genome from this study contained fermentation pathways involving the degradation of amino acids such as glycine, threonine, and lysine, suggesting that *Kyphosus* gut microbiota do not rely directly on dietary proteins for energy. Such lessened reliance on nitrogen-based substrates for fermentation is consistent with a low-protein, algae-based diet rich in available polysaccharides and limited in available nitrogen.

### Functional adaptations to life in the *Kyphosus* gut

**Fig 4:**
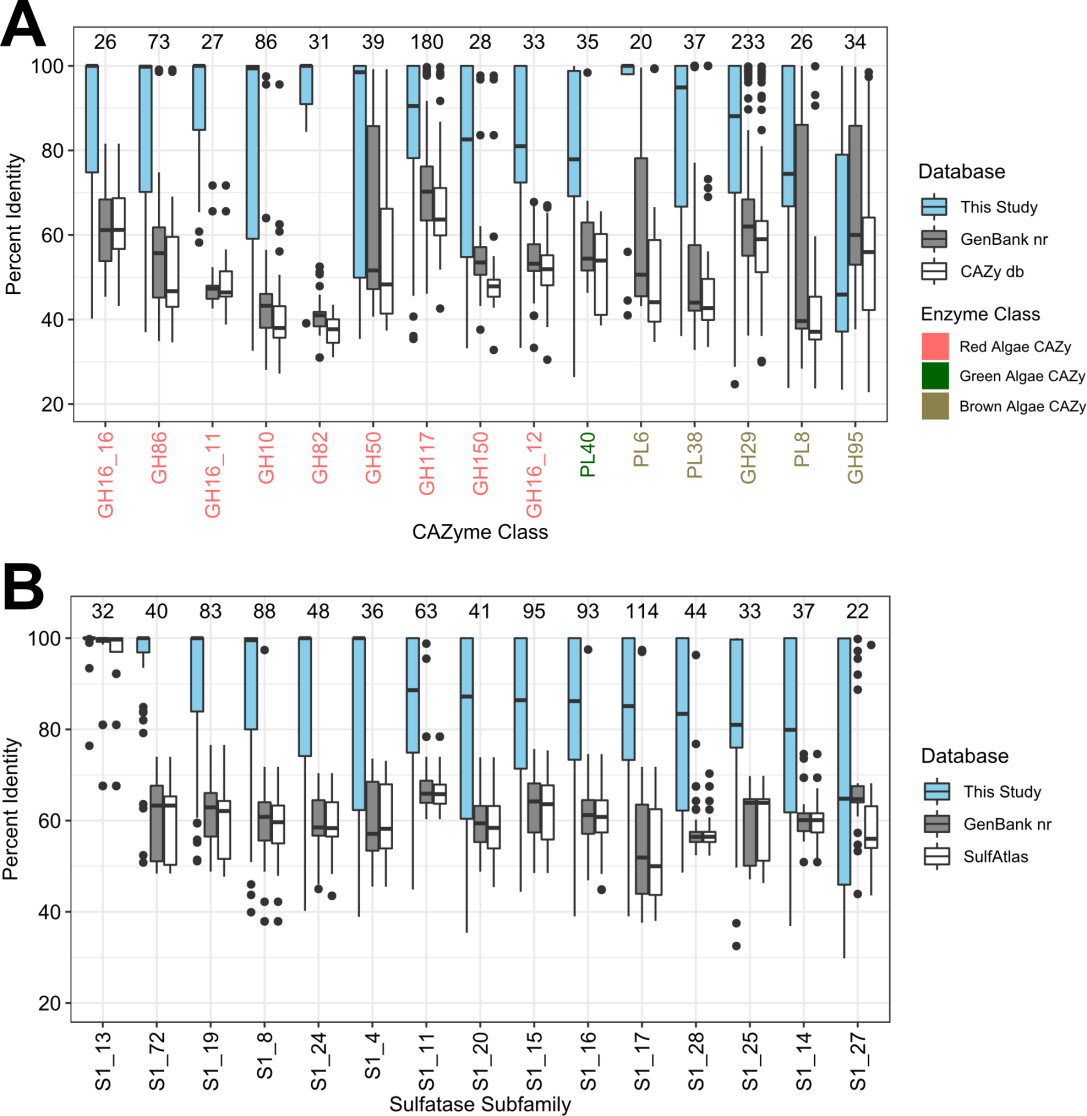
*Kyphosus* gut symbionts encode CAZymes and sulfatases divergent from other data sets and environments. Percent identity of binned (**A**) CAZymes and (**B**) sulfatases to best blast matches found in the following databases: all genes from MAGs in this study (blue), the GenBank nr database (gray), and either (**A**) the CAZy database or (**B**) the SulfAtlas database (white). CAZyme classes are colored based on the degradation of red, green, or brown algal polysaccharides. Each group is labeled by the number of genes with that enzyme annotation found in our MAGs.

Adaptations to environmental conditions in herbivorous fish gut microbes are reflected in the high abundance of CAZyme classes specifically targeting algal polysaccharides (20). Figure 4A shows that the amino acid sequences of CAZyme classes abundant in the MAGs of this study are well conserved across *Kyphosus* gut symbiont genomes. However, such enzymes are poorly represented in both specialty and general databases of previously described sequences, with closest enzyme homologs averaging less than 60% sequence identity for most of the highlighted CAZyme classes. Similar trends are observed for the sulfatase subclasses in *Kyphosus* gut symbiont genomes (Fig. 4B). Both cases demonstrate the extent that this study expands known sequence diversity within these enzyme classes, underscoring unusual domains that may not be captured by current databases.

**Fig 5:**
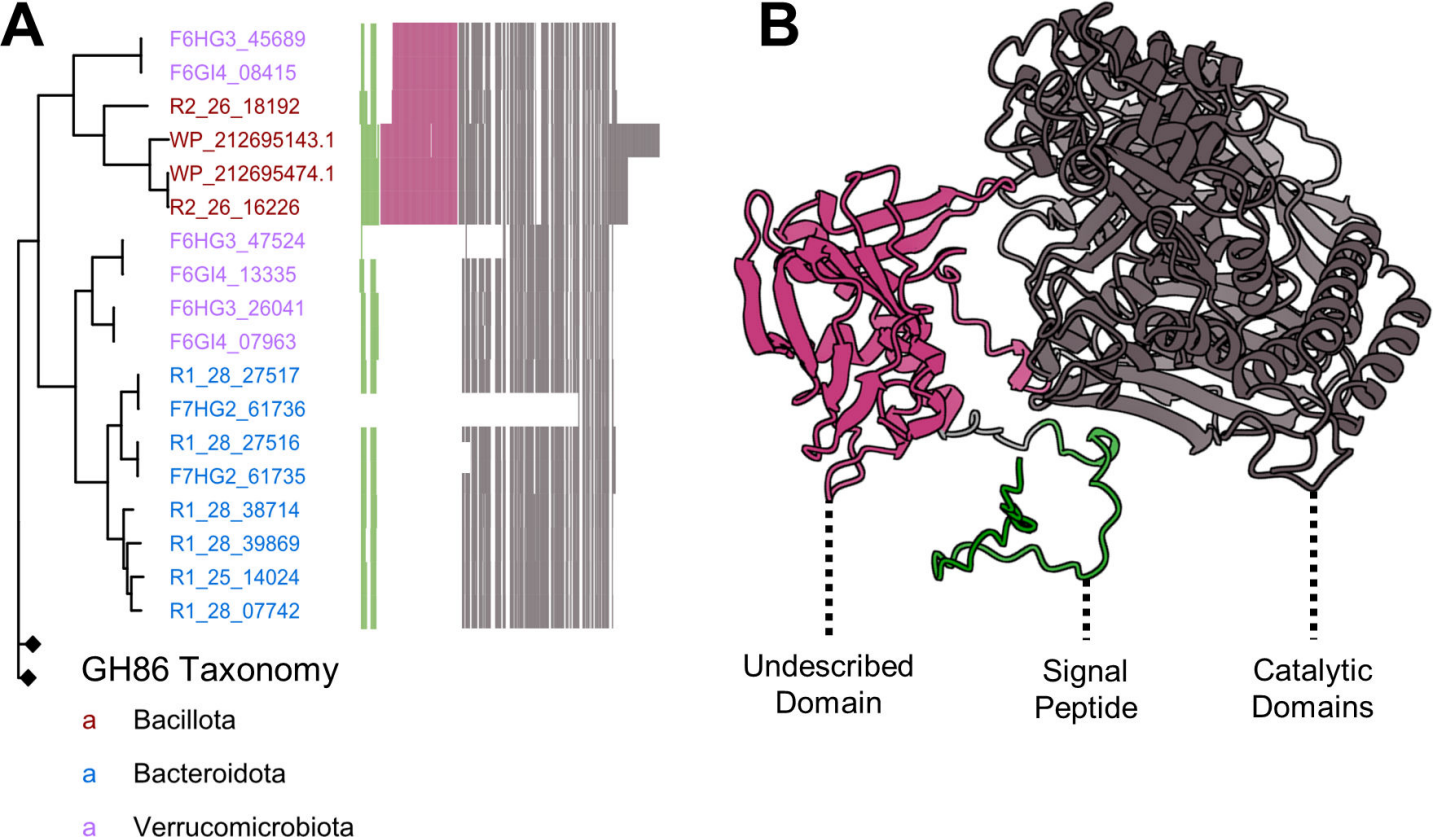
A β-agarase/β-porphyranase gene tree highlights an undescribed protein domain present in multiple phyla. (**A**) A gene tree of binned GH86 enzymes, with gene names colored by genome taxonomy. Nodes with black diamonds represent collapsed outgroup clades lacking the extra domain. A multiple sequence alignment is appended to the right of the tree, with colored vertical lines representing conserved amino acid positions and white vertical lines representing gaps. (**B**) The predicted protein structure of GH86 enzyme R2_26_16226, with conserved CAZy domains highlighted in gray, the predicted signal peptide in green, and the conserved new domain in pink. An uncollapsed version of the gene tree is included in Fig. S2, and a motif logo of the domain is presented in Fig. S3.

The discovery of novel enzyme sequences in these enzyme classes presents numerous opportunities to expand our understanding of marine polysaccharide degradation. One example using the phylogeny of CAZy class GH86, consisting of β-agarases and β-porphyranases, illustrates previously unappreciated cryptic variability within this enzyme family. A gene tree of class GH86 CAZyme examples from this study plus closest GenBank homologs (Fig. 5A) shows that many of these genes are associated with *Bacteroidota*, consistent with the high abundance of CAZymes and sulfatases found among MAGs from this phylum in *Kyphosus* guts (Fig. 2). Binned MAG genes annotated as β-porphyranases all originate from hindgut or enrichment samples, consistent with previously reported physiological localization of polysaccharide degradation capabilities (20). Surprisingly, two GH86 genes recovered in *Bacillota* MAGs from bioreactor enrichments and two *Bacillota* homologs from the NCBI nr database nested within a clade of genes from phylum *Verrucomicrobiota*. This unexpected pattern of association between genes from very distant microbial taxa has not been described in prior literature and may be indicative of horizontal gene transfer.

Amino acid insertions in this unique clade might either extend the signal peptide or contribute additional catalytic functionality (82). Among NCBI nr homologs, only genes from the hydrothermal vent genome *Vallitalea pronyensis* (WP_212695143.1 and WP_212695474.1) (83) contained this pattern of approximately 168 amino acids. No other entries in the GenBank nr database contained sequences matching this region at greater than 50% amino acid identity (Fig. S2). Outside of the clade containing this novel domain, variability occurs primarily in the putative signal peptide region at the N-terminus of the protein, while the downstream porphyranase domain itself is far more conserved. Figure 5B displays the predicted three-dimensional structure of a *Kyphosus* symbiont GH86 enzyme, with the additional uncharacterized region positioned between the predicted signal peptide and annotated catalytic β-agarase and β-porphyranase domains. Although the function of this domain cannot be determined bioinformatically, it provides an interesting subject for further enzymatic characterization. Potentially novel properties might include modified substrate specificity, substrate concentration dependence, catalytic efficiency, and/or tolerance of different abiotic conditions.

MAG sequences were interrogated using antiSMASH BGC detection software to determine whether *Kyphosus* gut-associated microbes might encode any unusual secondary metabolites. The majority of *Bacillota*, *Bacteroidota*, *Verrucomicrobiota*, and *Gammaproteobacteria* MAGs from both fish gut inocula and bioreactor enrichments encoded BGCs typical of taxonomic relatives found in other vertebrate gut environments, such as lanthipeptides, beta-lactones, and arylpolyenes (84, 85). However, BGCs were not particularly abundant in our MAG catalog relative to other similar genomes. Our recovered *Gammaproteobacteria*, *Bacillota*, and *Bacteroidota* average fewer BGCs per genome than a random set of seawater MAGs representing each taxonomic group from the OceanDNA database. Thus, our host-associated MAGs may contain fewer BGCs per genome than their free-living relatives.

**Fig 6:**
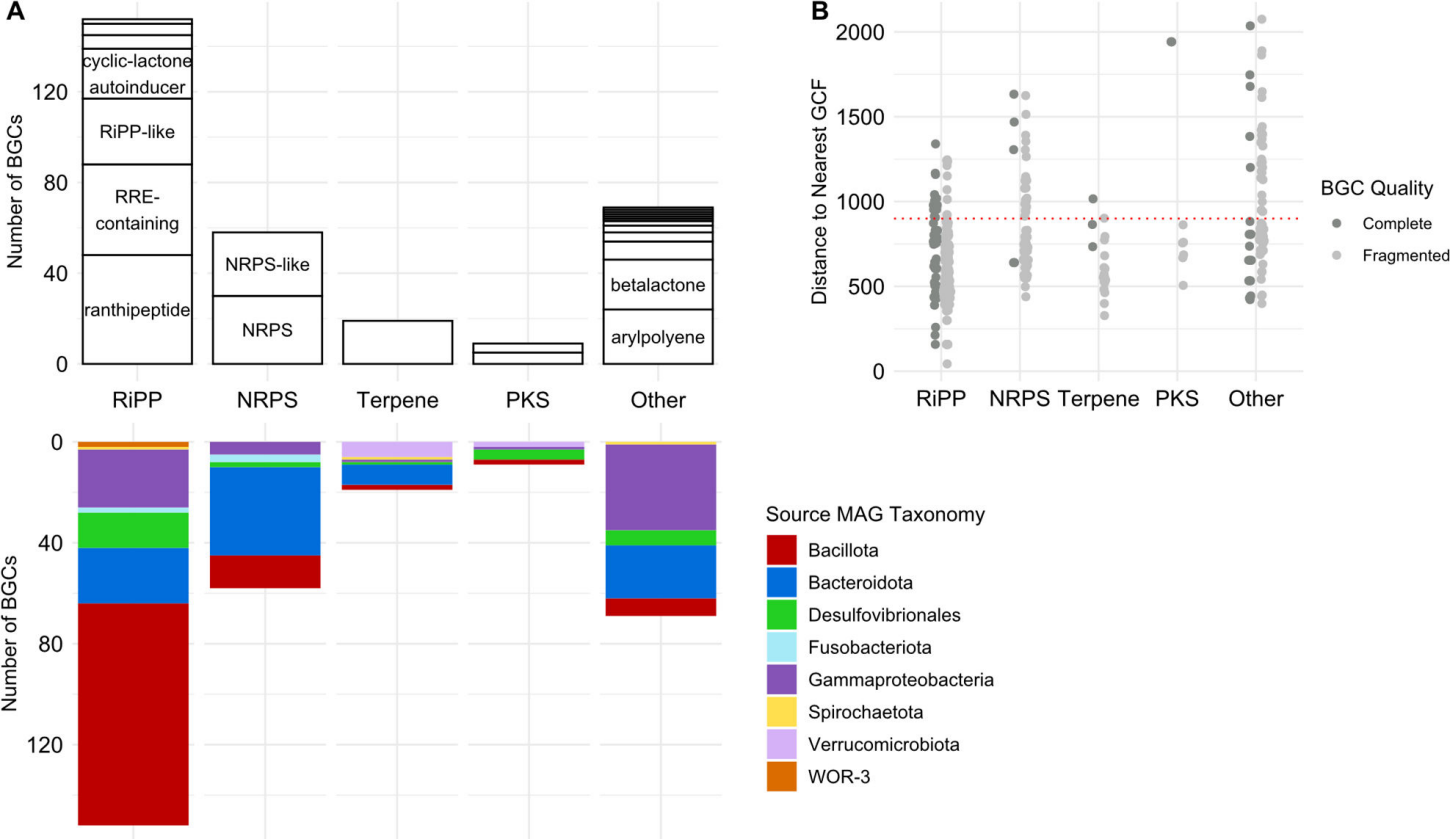
*Kyphosus* gut symbiont MAGs encode novel BGCs. (**A**) On the positive y-axis, counts of binned BGCs are grouped by BiG-SLiCE class and labeled by predicted product. On the negative y-axis, counts of binned BGCs are grouped by BiG-SLiCE class and colored by associated MAG taxonomy. (**B**) Distance of binned BGCs to the nearest gene cluster family as determined by BiG-FAM. A distance above 900, marked by a dashed red line, suggests novelty and divergence from previously described gene cluster families. BGCs are colored dark grey if they are annotated as complete by BiG-FAM. Abbreviations: RiPP, ribosomally synthesized and post-translationally modified peptide; RRE, RiPP recognition element; NRPS, non-ribosomal peptide synthetase; PKS, polyketide synthase.

A total of 307 BGCs were annotated within our MAGs (Fig. 6). Twenty-three annotated BGCs were determined to be complete, meaning they were not located on contig edges, based on BiG-FAM analysis of antiSMASH predictions (61). Twenty BGCs represent putative novel gene cluster families, with BiG-FAM distances exceeding the standardized cutoff score of 900 (Fig. 6B). These novel gene cluster families may represent unique natural products or enzymes specialized to the *Kyphosus* gut environment. Complete biosynthetic gene cluster annotations, novelty assessment, and associated taxonomy are included in Table S6.

### Community digestion of complex algal polysaccharides

**Fig 7:**
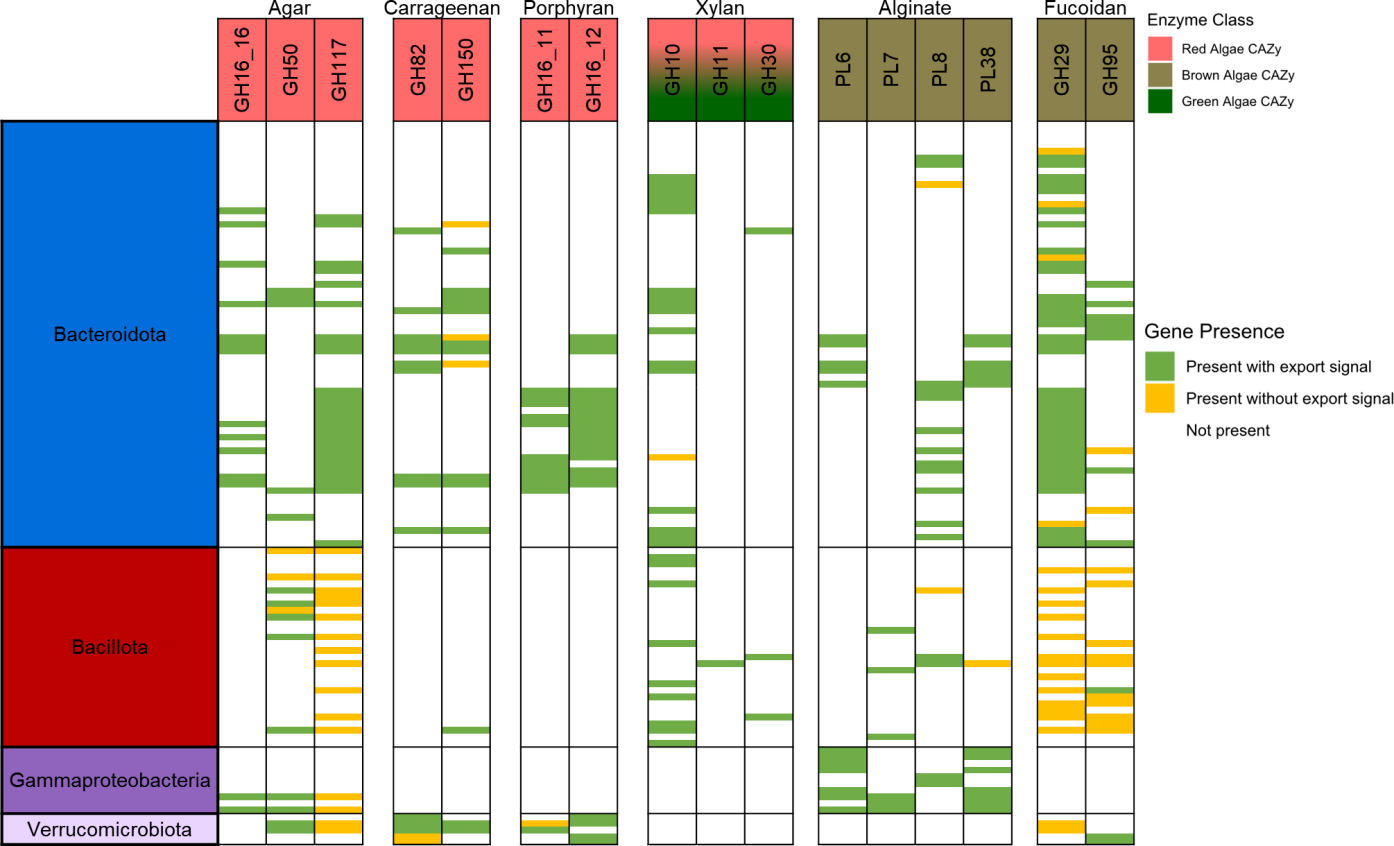
*Kyphosus* gut symbiont MAGs encode the capacity to degrade various algal polysaccharides collaboratively but not solitarily. Each row represents a single MAG from the annotated taxonomic lineage. Only MAGs from the four lineages with the highest concentration of CAZymes (*Bacillota*, *Bacteroidota, Gammaproteobacteria,* and *Verrucomicrobiota*) are shown. MAGs with no applicable CAZy classes are not shown, and CAZy classes not associated with a single substrate or not found in any MAG are not shown. Green bars denote a signal peptide annotated to at least one of the appropriate CAZyme in a single MAG, while yellow bars mark the absence of a signal peptide on all appropriate CAZyme candidates within a MAG.

Polysaccharide digestive capabilities vary among MAGs from different microbial taxa in the *Kyphosus* fish gut community, as shown in Fig. 7. Despite overall microbiome-wide diversity, the MAGs generated in this study show that few individual genomes contain all of the enzymes necessary to completely degrade even a single type of complex algal polysaccharide, let alone the huge variety of natural variants characteristic of marine macroalgae (86) that might be ingested by generalist herbivorous fishes. Each microbial genome instead contains a limited assortment of enzymes capable of partially degrading a selection of different carbohydrate moieties, including potentially incomplete breakdown products generated by other microbes. Combined pangenomic capabilities of several taxonomic groups appear to contain complementary collections of exported CAZymes that might facilitate adaptation to unpredictable variability in available polysaccharide content. Figure 7 summarizes predicted macroalgal digestion capabilities observed within individual MAGs for the most abundant taxonomic groups. The collaborative potential for all MAGs within each metagenomic sample is illustrated in Fig. S4.

Potential contributions to shared, community-wide degradation of algal polysaccharides through extracellular enzymes vary according to both microbial cell taxonomy and targeted substrate. More than 90% of CAZymes that target macroalgal polysaccharides from *Bacteroidota* MAGs contain signal peptides that indicate export or integration into the cellular membrane. In contrast, CAZymes in *Bacillota* MAGs largely lack these signal peptides in enzymes predicted to degrade fucoidan and agar but have more abundant signal peptides within the smaller set of CAZymes targeting xylan and alginates. Few *Bacillota* MAGs contain all of the enzymes required to fully degrade complex algal polysaccharides such as porphyran, suggesting that cells from this taxonomic group might scavenge partial breakdown products degraded extracellularly by other taxa.

*Verrucomicrobiota* polysaccharide digestion enzymes appear to be more specialized toward red algae, with genomes consistently containing CAZymes predicted to digest agar, carrageenan, and porphyran. However, MAGs from this phylum seem to be lacking enzymes predicted to target green or brown algal polysaccharides. *Gammaproteobacteria* MAGs appear to have more enzymes involved in the digestion of non-sulfated polysaccharides, such as alginate, and occasionally enzymes involved in agar degradation. Thus, the *Gammaproteobacteria* symbionts analyzed here may have specialized in polysaccharide types that are easier to digest.

## DISCUSSION

The recovery and characterization of 211 MAGs from *Kyphosus* gut and enrichment metagenomes connect detailed taxonomic classification with the potential of the major microbial contributors to digest complex algal polysaccharides. Algal polysaccharide-targeting enzymes from this study are divergent in sequence from previously sequenced and characterized representatives from other environments, clarifying prior assumptions about the metabolic capacities of this system using 16S rRNA or community composition. This study confirms and expands upon earlier work showing that certain members of the *Bacillota* and *Verrucomicrobiota* lineages are unexpectedly richer in some CAZyme and sulfatase enzyme classes than their respective taxonomic relatives (20). These CAZyme-rich MAGs provide the first genomic evidence supporting prior observations of laminarin, carrageenan, and alginate degradation in *Kyphosus* guts (87, 88). Differences between source inocula and the metagenomes of bioreactor enrichments starting from *Kyphosus* gut bacteria highlight potential challenges in harnessing this microbiota for bioenergy preprocessing of macroalgal feedstocks.

This study describes specific genes encoding SCFA production pathways in the genomes of fish gut microbiota. Microbial fatty acids serve as a key metabolite in gut-brain communication (89) and are a major source of available carbon for the host (90). SCFA pathway diversity is unexpectedly low for a system previously shown to contain high SCFA concentrations *in vivo* (16). However, this observation is consistent with a few dominant lineages, primarily the *Bacteroidota*, producing high amounts of SCFAs from the breakdown products of algal polysaccharides. Prior chemical work has observed that propanoate is more abundant than butyrate in *Kyphosus* guts (16), and our pathway enzyme abundance information at the genome level supports these observations (Fig. 3). Metabolic capacities in our *Kyphosus* metagenomes also match previous observations that bony fishes with carbohydrate-rich diets consistently lack branched SCFAs and have low rates of protein fermentation by gut bacteria (91).

Mannitol has been suggested as a major source of fermentation substrate in some algivorous fishes, based on the large percentage of mannitol in some brown macroalgae, observed degradation of mannitol by *Kyphosus* guts (92), and the relative accessibility of this compound compared to complex sulfated algal polysaccharides. The presence of both mannitol 2-dehydrogenase (EC 1.1.1.67) and D-mannitol-1-phosphate dehydrogenase (EC 1.1.1.17) genes in our MAGs, the latter of which was initially proposed by Seeto et al. (93) but not found in recent metagenomic investigations of *Kyphosus* guts by Stevenson et al. (19), suggests that mannitol utilization may differ more between individual fish and algivorous species than previously thought.

Other observations in prior work on *Kyphosus* (16) noted rates of sulfate reduction higher than methanogenesis, although both processes were negligible compared to SCFA production. This aligns with the low abundance of *Desulfovibrionales* and the near complete absence of Archaea in our metagenomes, consistent with observations that dietary red macroalgae inhibit methanogenesis and thus the success of gut Archaea (33). Both sulfate reduction and methanogenesis appear to be minor sources of energy available for Kyphosid host absorption, compared to fermentation by *Bacteroidota* and *Bacillota*.

Although *Kiritimatiellales* MAGs recovered from *K. cinerascens* fish guts contain more enzymes targeting algal polysaccharides than other members of their phyla, these taxa were not recovered from enrichment metagenomes. However, this should not be problematic for enrichment processing if the dominant *Bacteroidota* contain CAZymes with overlapping specificities for the same substrates, as suggested in Fig. 7. Additional work comparing MAGs from lumen and mucosal samples may provide additional insights into metabolic capacities that might be more abundant in the transient vs permanent resident fraction of the microbiome (19). Future enzyme-focused work will be needed to characterize sample-specific polysaccharide degradative chemistry in order to parse the specific roles of each taxa. *Vallitalea* and *Verrucomicrobiota* enzymes may encode some unique functionalities, as suggested by the extra domain present in their β-porphyranase sequences (Fig. 5). Isolation and *in vitro* characterization of bioinformatically predicted enzyme activities will be necessary to fully integrate these discoveries into aquaculture and bioenergy applications.

Metagenomic data from the MAGs in this study suggest that few individual cells have the genomic potential to independently degrade all of the complex sulfated polysaccharide substrates present in marine macroalgae. However, secreted and extracellularly exposed transmembrane CAZymes may enable collaborative interactions between fish gut microbes to facilitate complete digestion of these molecules, without the high metabolic cost of encoding a complete, independent repertoire in every genome. A division of labor strategy cannot be fully confirmed without *in vitro* tests (94), although the first condition of genomically encoded functional complementarity appears to hold true between *Kyphosus* symbionts based on bioinformatic criteria. In one similar study, gene-based observations of complementarity for marine lignocellulose-degrading bacteria align with *in vitro* observations that support a division of labor hypothesis (95). Future work involving cultured representatives and enriched microcosms will be required to pin down the ecological strategies used by symbionts in this system.

This study provides a new baseline for *Kyphosus* microbiota at the genome level but begets many new questions requiring additional experimentation. Further work that connects enrichment composition, feedstock polysaccharide composition, and physical configuration parameters to chemical measurements of degraded polysaccharides will help determine which phyla are required for complete polysaccharide breakdown. The incorporation of novel enzyme sequences identified here may warrant the creation of new subclasses, based on classification techniques such as sequence similarity networks (96). Isolation and characterization of divergent proteins with unexpected new domains may reveal new enzymatic properties unique to this system. Metatranscriptomic analyses utilizing the genome catalogs presented here will enable detailed analysis of substrate-specific metabolic pathway expression and species collaboration. *Kyphosus* digestive systems have long been studied as models for herbivorous fish gut fermentation and can now be explored further using these additional techniques to deliver a deeper understanding of their degradative and fermentative capabilities.

### Conclusion

The new metagenome-assembled genomes recovered from herbivorous fish guts and corresponding bioreactors described here provide a genomic catalog of *Kyphosus* gut symbionts highlighting untapped diversity in enzymatic and collaborative potential in the degradation of algal polysaccharides. The extensive sequence divergence of enzymes encoded within these genomes from previously characterized CAZyme family examples supports the promise of herbivorous fish guts as a source of novel and industrially relevant enzymes. Expansion of these discoveries will not only clarify ecological interactions but have the potential to improve the applicability of macroalgae in the bioenergy and aquaculture sectors.

## Data Availability

All custom code used for data analysis and visualization are available at https://github.com/AaronAOliver/KyphosusMAGs. Sequence reads are available under SRA BioProject numbers PRJNA819194 and PRJNA1023379. Complete MAG sequences and predicted proteins are available on Zenodo (https://zenodo.org) under DOI no. 10.5281/zenodo.8277654.

## SUPPLEMENTAL MATERIAL

Supplemental figures mbio.00496-24-s0001.pdf Fig. S1 to S4.

Table S1 mbio.00496-24-s0002.xlsx Enrichment sample information.

Table S2 mbio.00496-24-s0003.xlsx Assembly statistics for enrichment metagenomes.

Table S3 mbio.00496-24-s0004.xlsx MIMAG-compliant completeness and lineage information for recovered MAGs.

Table S4 mbio.00496-24-s0005.xlsx Predicted viral contigs and auxiliary metabolic genes.

Table S5 mbio.00496-24-s0006.xlsx Mannitol utilization genes for MAGs and metagenomes.

Table S6 mbio.00496-24-s0007.xlsx Detailed BGC annotations and nearest gene cluster families.

ASM does not own the copyrights to Supplemental Material that may be linked to, or accessed through, an article. The authors have granted ASM a non-exclusive, world-wide license to publish the Supplemental Material files. Please contact the corresponding author directly for reuse.
