# Supplementary material for: Enrichable consortia of microbial symbionts degrade macroalgal polysaccharides in Kyphosus fish
Source: mBio. 2024 Mar 27;15(5):e00496-24. doi: 10.1128/mbio.00496-24 (PMC11077953; doi:10.1128/mbio.00496-24)
Supplement: Supplemental figures — Fig. S1 to S4. [file mbio.00496-24-s0001.pdf]

Supplementary Figures

Enrichable consortia of microbial symbionts degrade macroalgal polysaccharides in *Kyphosus* fish

Aaron Oliver, Sheila Podell, Linda Wegley Kelly, Wesley J. Sparagon, Alvaro M. Plominsky, Robert S. Nelson, Lieve M. L. Laurens, Simona Augyte, Neil A. Sims, Craig E. Nelson, Eric E. Allen

Supplementary Figure S1. Recovered MAGs from fish gut and enrichment metagenomes. Enrichment samples on the left half of the figure are labeled with inoculant fish taxa, gut region, and bioreactor feed. Wild fish gut metagenomic samples on the right half of the figure were binned from metagenomes previously assembled by Podell et al. (20). Abbreviations: GI, midgut; HG, hindgut; medley, a combination of *Ulva*, *Sargassum*, and *Agardhiella* seaweed.

Figure S2. Complete gene tree with all binned GH86 CAZymes. Phylogenetic tree with gene names colored by genome taxonomy. Cells mark the source of each CAZyme and whether SignalP predicts the presence of a signal peptide.

Figure S3. Motif logo of previously undescribed residue pattern for GH86-associated domain. Logo created using WebLogo. Residue numbers are based on position in the pattern, rather than the parent protein. Few residues are conserved across all representatives.

Figure S4. *Kyphosus* gut symbiont MAGs encode the capacity to degrade various algal polysaccharides collaboratively. Each row represents all the MAGs from the corresponding metagenome. Green bars denote a signal peptide annotated to at least one of the appropriate CAZyme in a single MAG, while yellow bars mark the absence of a signal peptide on all appropriate CAZyme candidates within the MAGs of a metagenome.

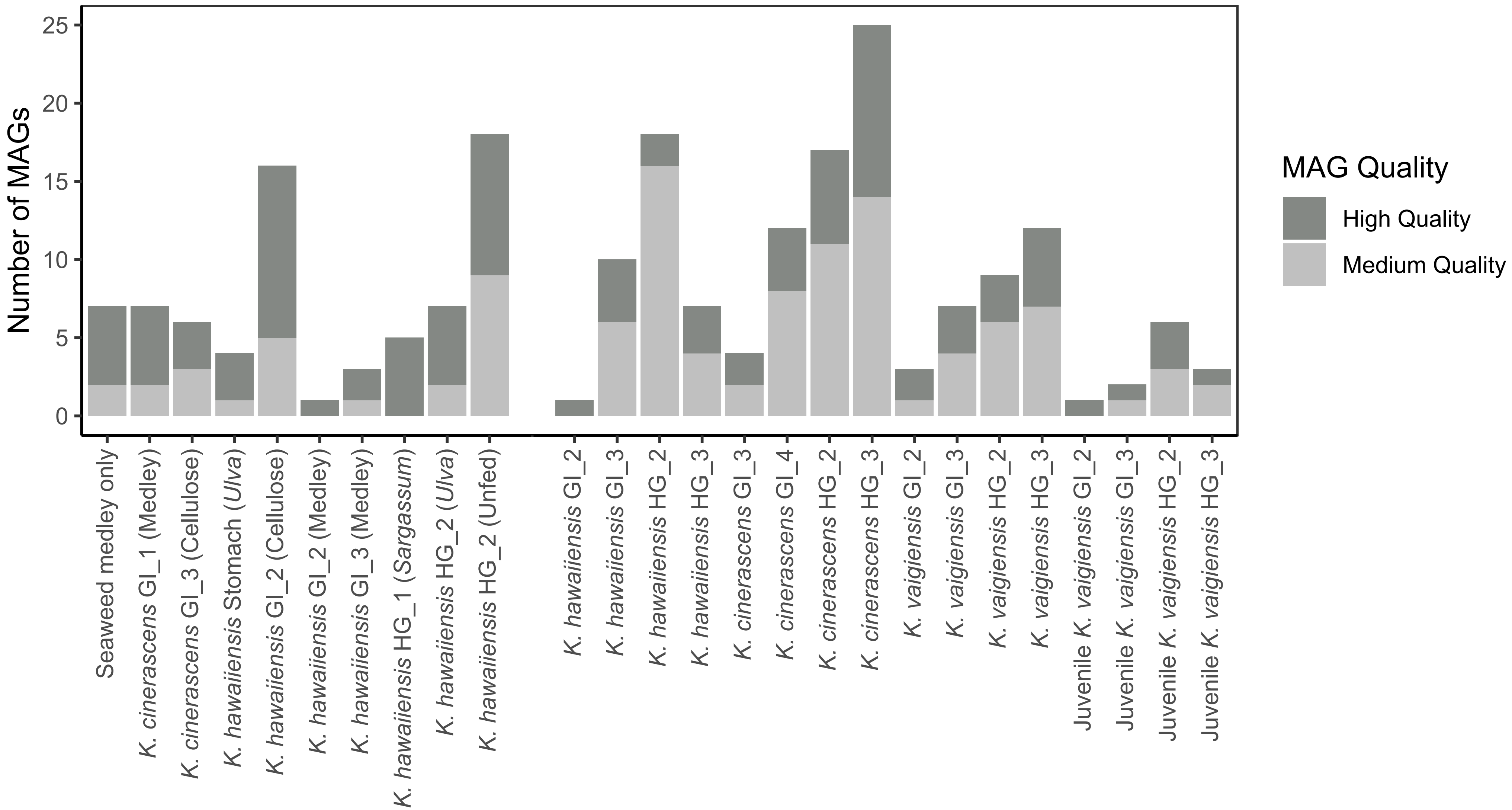

Supplementary Figure S1. Recovered MAGs from fish gut and enrichment metagenomes. Enrichment samples on the left half of the figure are labeled with inoculant fish taxa, gut region, and bioreactor feed. Wild fish gut metagenomic samples on the right half of the figure were binned from metagenomes previously assembled by Podell et al. (20). Abbreviations: GI, midgut; HG, hindgut; medley, a combination of *Ulva*, *Sargassum*, and *Agardhiella* seaweed.

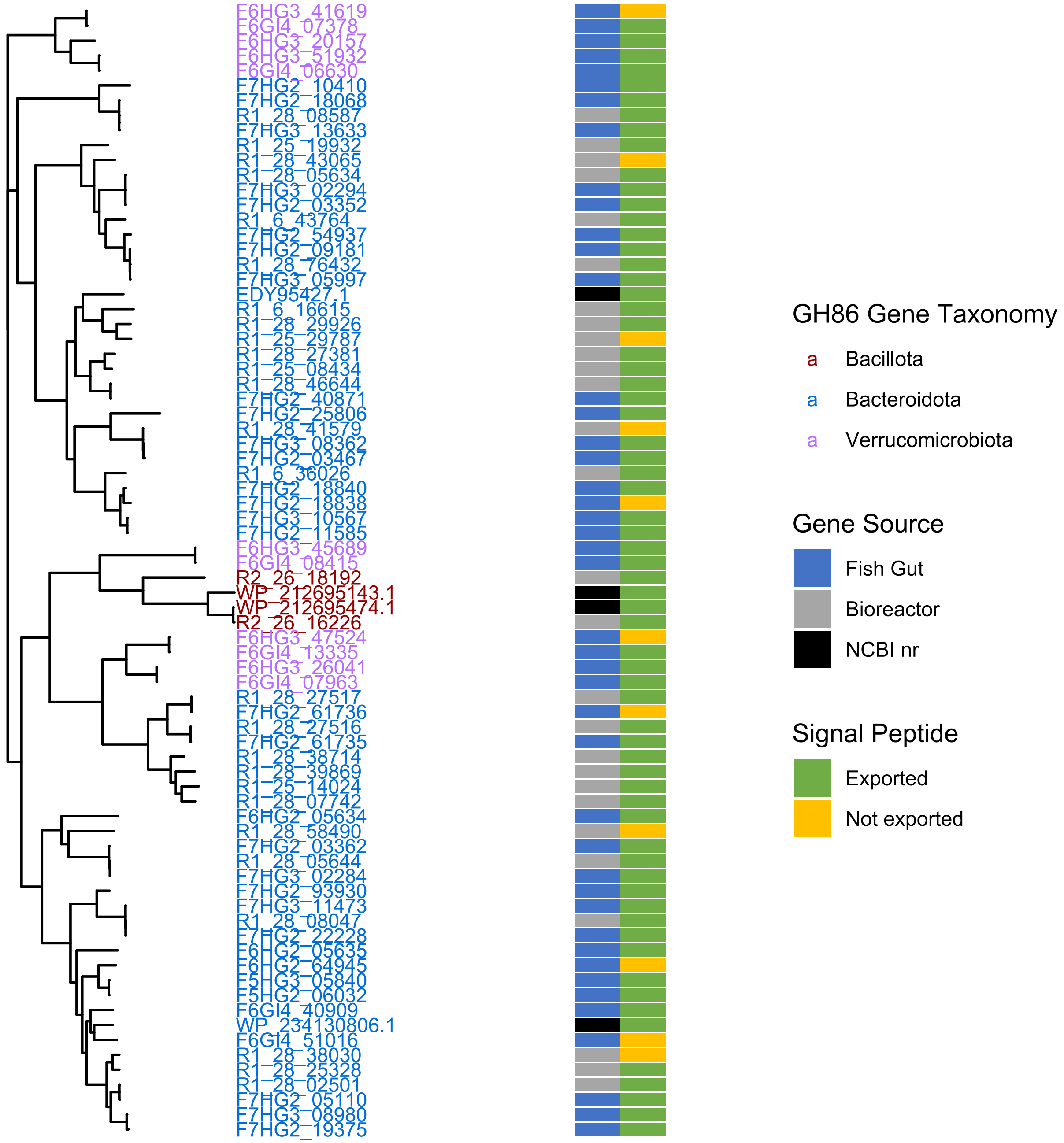

Supplementary Figure S2. Complete gene tree with all binned GH86 CAZymes. Phylogenetic tree with gene names colored by genome taxonomy. Cells mark the source of each CAZyme and whether SignalP predicts the presence of a signal peptide.

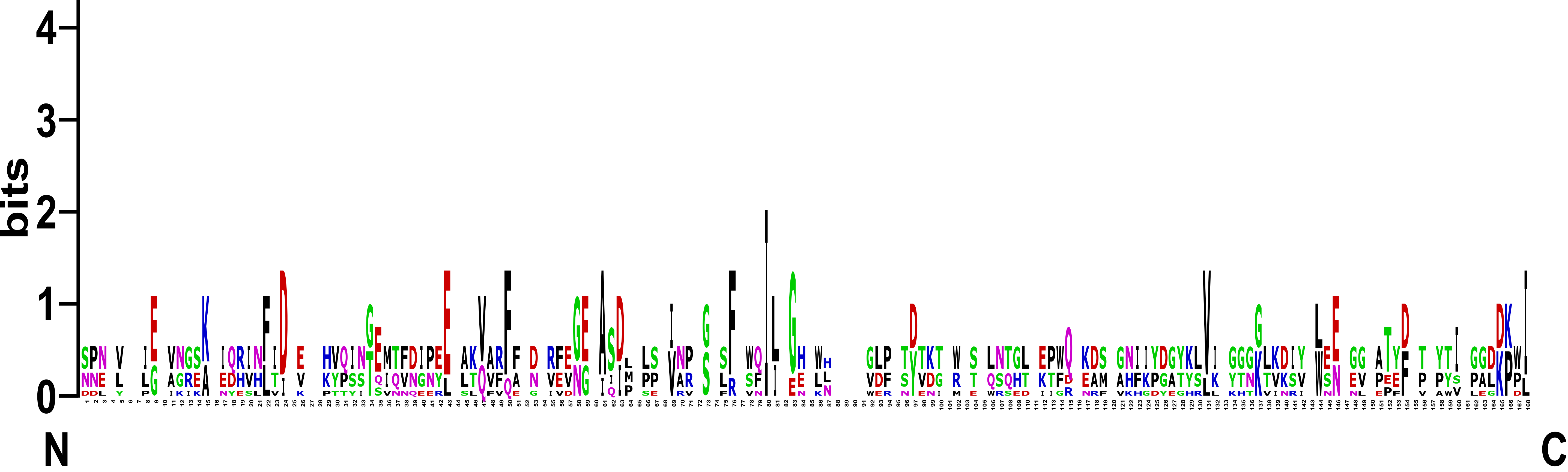

Supplementary Figure S3. Motif logo of previously undescribed residue pattern for GH86-associated domain. Logo created using WebLogo. Residue numbers are based on position in the pattern, rather than the parent protein. Few residues are conserved across all representatives.

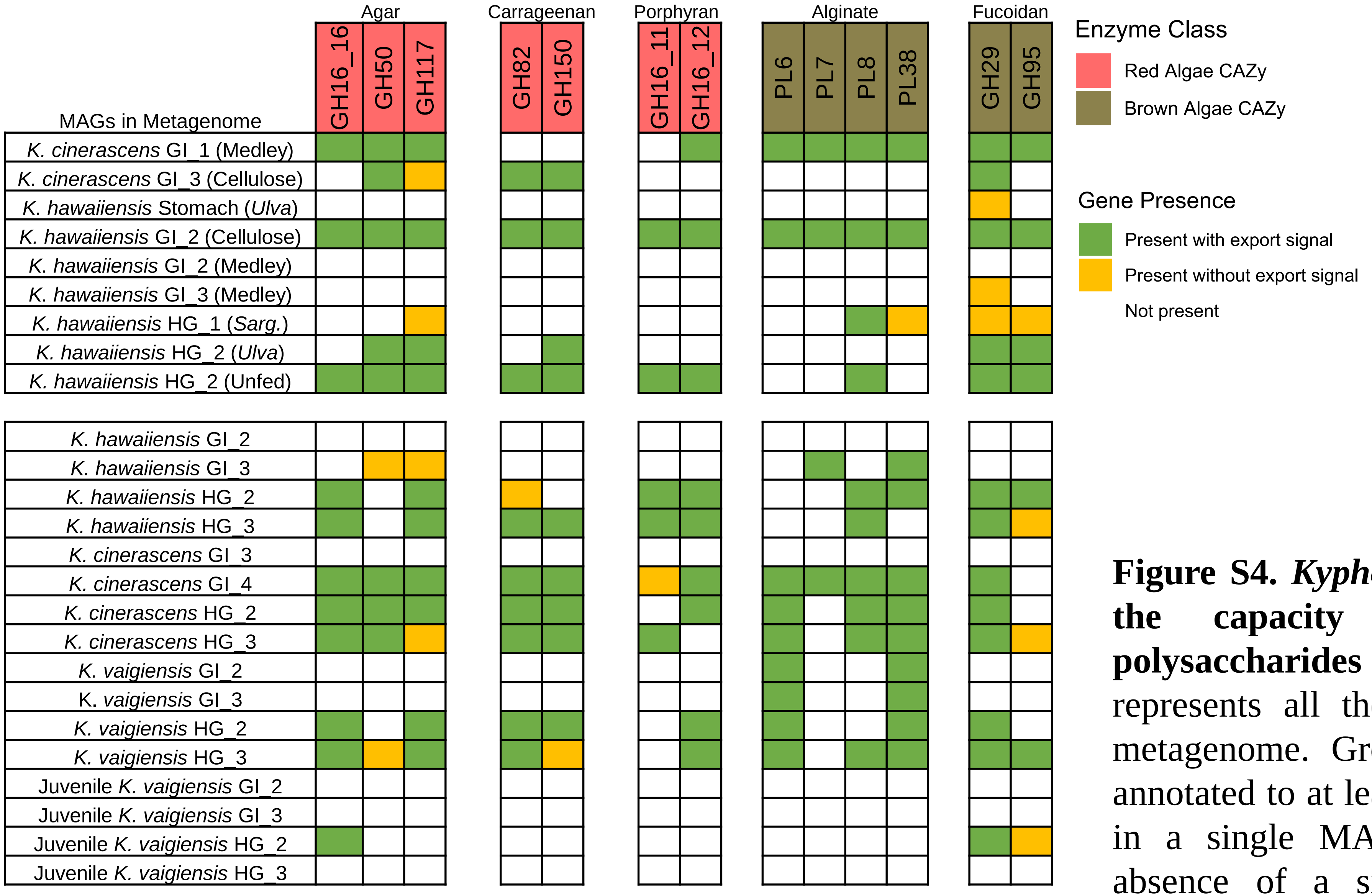

Figure S4. *Kyphosus* gut symbiont MAGs encode the capacity to degrade various algal polysaccharides collaboratively. Each row represents all the MAGs from the corresponding metagenome. Green bars denote a signal peptide annotated to at least one of the appropriate CAZyme in a single MAG, while yellow bars mark the absence of a signal peptide on all appropriate CAZyme candidates within the MAGs of a metagenome.
